# Extracellular Vesicles in Oral Squamous Cell Carcinoma and Oral Potentially Malignant Disorders: A Systematic Review

**DOI:** 10.3390/ijms21041197

**Published:** 2020-02-11

**Authors:** Tami Yap, Neha Pruthi, Christine Seers, Simone Belobrov, Michael McCullough, Antonio Celentano

**Affiliations:** Melbourne Dental School, The University of Melbourne, 720 Swanston Street, Carlton, VIC 3053, Australia; npruthi@student.unimelb.edu.au (N.P.); caseers@unimelb.edu.au (C.S.); belobrov@student.unimelb.edu.au (S.B.); m.mccullough@unimelb.edu.au (M.M.); antonio.celentano@unimelb.edu.au (A.C.)

**Keywords:** oral squamous cell carcinoma, extracellular vesicles, exosomes, oral premalignant lesions, mouth neoplasms, microvesicles, oral potentially malignant disorders

## Abstract

Extracellular vesicles (EVs) are secreted from most cell types and utilized in a complex network of near and distant cell-to-cell communication. Insight into this complex nanoscopic interaction in the development, progression and treatment of oral squamous cell carcinoma (OSCC) and precancerous oral mucosal disorders, termed oral potentially malignant disorders (OPMDs), remains of interest. In this review, we comprehensively present the current state of knowledge of EVs in OSCC and OPMDs. A systematic literature search strategy was developed and updated to December 17, 2019. Fifty-five articles were identified addressing EVs in OSCC and OPMDs with all but two articles published from 2015, highlighting the novelty of this research area. Themes included the impact of OSCC-derived EVs on phenotypic changes, lymph-angiogenesis, stromal immune response, mechanisms of therapeutic resistance as well as utility of EVs for drug delivery in OSCC and OPMD. Interest and progress of knowledge of EVs in OSCC and OPMD has been expanding on several fronts. The oral cavity presents a unique and accessible microenvironment for nanoparticle study that could present important models for other solid tumours.

## 1. Introduction

Oral squamous cell carcinoma (OSCC) is a highly debilitating disease that is often fatal. Early stage OSCC has the most favourable prognosis and requires less aggressive treatment; however, more than half of patients present with an advanced disease [1]. Advances in the early identification and improved targeted treatment to reduce morbidity continue to be sought. An area that presents an opportunity for the early identification of OSCC is the study of oral potentially malignant disorders (OPMD), considered a group of disorders that precede OSCC development, which can be visualized and assessed clinically. The oral cavity presents a unique and accessible environment to source nano-sized particles for study of the presence of OSCC and OPMD.

Extracellular vesicles (EVs), composed of an outer lipid bilayer containing proteins and nucleic acids, have been identified as an important means of cell-to-cell communication, both near and distant, in the body [2]. Tumour derived EVs can impact cellular processes of effector cells within the tumour environment as well as at distant sites, creating favourable environments for tumour growth and spread [3]. A specific type of EV are exosomes containing nucleic acids, proteins and lipids that appear to be purposefully packaged. This is exemplified by the observable differences between exosome-contained cargo, such as non-coding microRNA with microRNA from donor cells, suggesting a key way that tumour cells can influence their surrounding microenvironment [4,5]. Interest is expanding in the use of biofluid EVs as a potential non-invasive source of prognostic and diagnostic biomarkers. Furthermore, fully understanding the role that EVs have in influencing the tumour phenotype, immune modulation and preparation of metastatic bed has the potential for the development of more sophisticated and targeted treatments.

EVs are a complex group of vesicles that originate from distinct subcellular compartments. Cells release many types of vesicle subpopulations such as exosomes, microvesicles, apoptotic vesicles, lipoproteins and chylomicrons. The two most characterised types of EVs have differing physiological and pathological functions: exosomes [6] derived from multi-vesicular bodies (MVBs), a specialised subset of endosomes that contain intra-luminal membrane-bound vesicles; and microvesicles or ectosomes [7] that are derived from the cytoplasmic membrane [8,9]. In addition to different sites of origins, the two types of EVs have differing intracellular life before discharge. It has now been well established that although the term “exosomes” has often been used in research articles for isolated EVs, most historical and novel purification protocols such as differential ultracentrifugation, filtration and precipitation kits, co-isolate different types of EVs [10]. Therefore, the 2018 minimal information for studies of extracellular vesicles (MISEV) guidelines recommend that unless specific markers of subcellular origin can be established, future authors are encouraged to use terms for EV subtypes that refer to physical characteristic, biochemical composition or description of conditions or cell of origin [11]. This will enable a more accurate determination of the contribution of cargoes of each EV subtype to a physiologic state.

In this systematic review, we present the current state of knowledge with respect to EV studies conforming with the MISEV 2018 guidelines that involve clinically defined OSCC and OPMDs and OSCC-derived cell lines.

## 2. Results

### 2.1. Study Selection and Characteristics

The primary literature search, as of 12 October 2018, identified 1247 papers, of which 801 were unique. Screening of these by title resulted in retention of 280 records and following abstract screening, 108 reports were retained for full text review. Finally, of the 108 articles screened, 32 articles were accepted by the reviewers for final inclusion. A second search performed on 17 December 2019, identified a further 23 articles eligible for inclusion and review. This indicates a rapid rise in interest in the field of EV analysis in relation to OSCC and OPMDs and/or a greater propensity for investigators to adhere to the MISEV guidelines.

Of the 55 retained studies, 43 included in vitro components, 12 included in vivo components, and 16 studies included clinical sample derived EV components in their design. There were 13 studies which compared EVs from clinical cohorts (Table 1) and 29 studies presenting novel findings of EVs derived from and on primary or established cell lines (Table 2).

**Figure 1:**
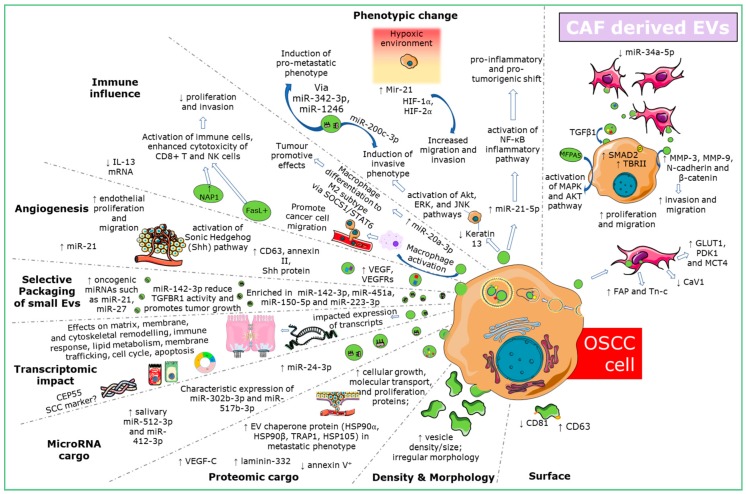
Known roles and interactions of EVs in OSCC.

Various EV isolation and purification techniques were reported with ultracentrifugation being the most common technique for EV isolation and immunoblotting for characterization and classification of EVs. The majority of the studies were conducted in China, Japan and the USA. The oldest study included in this review was from 2005 [12], with 53 of 55 articles published from 2015 onwards. The articles variously describe the EV morphologic characters and cargo and the role of specific molecules in EV-mediated and EV-employed influence from and on OSCC, OPMD and stromal cells types. The main features of OSCC- EVs have been graphically summarized in Figure 1.

### 2.2. Increased Abundance and Altered Morphology of OSCC-Derived EVs

Several EV studies suggest morphological and or volume differences between EV isolated from healthy individuals and individuals with OSCC and following OSCC resection. These observations correlate with observations from other body fluids [52]. Using atomic force microscopy (AFM), Sharma et al. (2011) [13] showed that salivary exosomes from healthy patients (*n* = 5) had homogeneous and circular morphology, and had a significantly smaller size range than salivary exosomes from oral cancer (OC) patients (*n* = 5) (40–80 nm vs. 20–400 nm, *p* < 0.05). Furthermore, the exosomes in saliva of OC patients were 2- to 4-fold more abundant and had greater morphological variation in shape with aggregation and multivesicular bodies present [13]. Zlotogorski-Hurvitz et al. (2016) [15] also examined OC derived exosomes in unstimulated saliva isolated using ultracentrifugation. Initially they utilised nano-tracking analysis (NTA) to show that exosomes were of significantly higher concentration in the (pooled) oral fluids of OC patients (36.0 ± 7.5E8 particles/mL) than in the pooled oral fluid healthy individuals (17.9 ± 12.45E8 particles/mL) (*p* = 0.01). The NTA analysis also indicated that exosomes were significantly larger in size in the OC patient oral fluid pool samples (95.36 ± 36.76 nm, *n* = 11) than the healthy individual sample pool (49.05 ± 32.87 nm, *n* = 17) (*p* = 0.002) [15]. Pooling of the samples from the cancer and healthy controls unfortunately precluded them from being able to determine the median exosome size distribution between the clinical types. NTA analysis was not performed on isolated exosomes due to difficulty in separating the tightly packed vesicles resulting from the ultracentrifugation. This observation would indicate that some caution should be shown towards “individual” exosome data derived from ultracentrifuged exosome pellets. In an alternative approach, bio-structural differences between salivary exosomes from OC patients and healthy individuals were assessed using Fourier-transform infrared (FTIR) spectroscopy, revealing 3 signal intensity ratios that were significantly increased in OSCC exosomes versus controls (I_1,404_/I_2,924_, *p* = 0.005), (I_1,033_/I_1,072_, (*p* = 0.024) and (I_2,924_/I_2,854_, *p* = 0.026) [17]. A developed discrimination function model enabled differentiation of salivary exosomes with a sensitivity of 100% and specificity of 89%, finding that early- and late-stage OSCC clustered together [17].

In contrast, Zhong et al. (2019) [19] who reported a rigorous saliva sample collection protocol, did not find that salivary microvesicles (MV) in samples sourced from 65 OSCC patients, 21 patients with an oral ulcer or 42 healthy donors differed in physical morphology, size distribution and zeta potential, as assessed by TEM, flow cytometry and dynamic light scattering. However, there were significantly higher levels of salivary MVs in the total cohort of OSCC patients compared to healthy individuals (*p* < 0.01) and patients with oral ulcers (*p* < 0.05). Furthermore, increased salivary MV abundances were correlated with metastasis and tumour stage, but discrimination between healthy donors and the early stage OSCC subgroup alone (Stage I + II) did not reach significance with this cohort. Circulating MVs were also found to be significantly higher in OSCC patients compared to healthy donors (*p* < 0.001), although no correlation in quantification between salivary and circulating MVs was identified [19]. However, no significant relationship was found between salivary MV levels and overall survival [19], and so although salivary MV abundance might serve as an indicator of disease presence and progression, further investigation is to understand its potential as needed to assess prognosis.

### 2.3. Differential Expression of OSCC-Derived EV Surface and Cargo Proteins

It has been reported that exosomes from OSCC patients showed an increase in CD63 surface density assessed by force spectroscopy compared to those from healthy controls, although this was not described statistically [13]. Increased EV surface expression, of either CD63 or CD9, was not found using ELISA, although CD81 expression was significantly lower in OC derived fluids (*p* = 0.032) [15].

Zorilla et al. (2019) [22] collected plasma samples from 10 OSCC patients with large tumours (T4) with no distant metastasis both pre- and shortly post-tumour and lymph node resection. Isolated plasma EVs were characterized for CD63 and CAV-1 using immunocapture-based assay. Overall, this pilot study did not report findings that reached statistical significance, but found trends that CD63 positive plasmatic EV levels decreased in the 7 days following surgery (*p* = 0.091), whereas CAV-1 levels increased (*p* = 0.237), most likely due to post-surgery inflammatory response. Further trends suggested poor survival associated with continuing high plasmatic CD63 EV levels following resection (*p* = 0.808) [22]. Expansion of a study of this type using a larger cohort may indicate if this approach has some promise as a prognostic survival indicator.

Using a shotgun proteomic approach with salivary EVs of healthy (*n* = 10) and OC (*n* = 7) subjects, Winck et al. (2015) identified 381 proteins by mass spectrometry with 18 proteins detected exclusively in the healthy group EVs, 4 proteins exclusively identified in the OC group EVs and 8 proteins differentially expressed (*p* < 0.05) between the groups [14]. Gene ontology analysis of these proteins revealed over-representation of antigen binding and enzyme inhibitory functions, proteins related to transport, particularly of metals, as well as cellular growth and proliferation [14].

Questioning evolution of EV proteomic cargo in OSCC metastasis, Ono et al. (2018) [53] have characterized the EV proteomes of an OSCC line (HSC-3) and a metastatic sub-line (HSC-3-M3) with particular interest in heat shock proteins as chaperone proteins. The metastatic OSCC cells secreted larger EVs than those derived from the parental OSCC cells, as measured by TEM (*p* = 0.045) and 32% of the proteome was different between the two cell lines. Of 192 EV proteins identified, 9 chaperones were at higher levels in HSC-3-M3 EVs as compared to HSC-3 EVs, including HSP90α, HSP90β, TRAP1 and HSP105, suggesting that these EV proteins may potentially be biomarkers of a metastatic phenotype [53].

Zhong et al. (2019) found that the abundance of vascular endothelial growth factor C (VEGF-C) in the tumour samples of OSCC patients correlated with patient levels of salivary MVs [19]. VEGF-C is one of the major pro-lymphangiogenic growth factors, thus correlating metastasis to lymph nodes with salivary MV levels. Interestingly, the percentage of annexin V^+^, as a marker of apoptotic cell MV origin, was approximately 50% in salivary MVs of both OSCC patients and healthy donors, whereas few circulating MV of OSCC patients were annexin V^+^. Furthermore, within the OSCC group, the percentage of annexin V^+^ MV was significantly decreased in salivary MVs of patients with a higher pathological grade (III) when compared to those with lower pathological grades (I and II) (*p* < 0.01), which was associated with poorer survival rates (*p* < 0.05) [19].

Serum exosomal protein content has been measured and explored as an indicator of locoregional spread. Li et al. (2019) [54] isolated exosomes from serum of subjects, 10 each with OSCC with lymph node metastasis (LNM), OSCC with no LNM (NLNM) and healthy controls (HC). Proteomic comparison using a combination of liquid chromatography-mass spectrometry and mass spectrometry followed by clustering into gene ontology categories. Of 415 proteins identified, 37 and 28 differentially expressed proteins were identified in OSCC-LNM compared to HC and OSCC-NLNM, respectively. Combined with qPCR-based validation findings from tissue, serum and whole blood samples, they highlighted that serum exosomal proteins, PF4V1, CXCL7, F13A1 and ApoA1 may be related to the OSCC lymph node metastasis [54]. ROC analysis using relative abundances of ApoA1, CXCL7, PF4V1 and F13A1 in serum, serum exosomes and whole blood indicated some potential application as novel predictive circulating biomarkers for OSCC with LNM but are not useful in prognosis. Expansion of the cohort size would test the validity of these conclusions.

Laminin-332 in cancer cells has been shown to promote cell growth, invasion and metastasis [55]. In accord with this, Wang et al. (2019) [44] found that Laminin-332 proteins (laminin α3, β3 and γ2) were upregulated in LN1-1 cell line EVs versus the levels found in the EV of the OEC-M1 cell line, from which LN1-1 cells were derived (>1.5 fold) and the number of laminin γ2-positive gold particles per EV was greater in LN1-1 EVs than in OEC-M1 EVs (*p* < 0.01) [44]. Comparing 10 healthy controls and 20 OSCC, ELISA detected a significantly higher level of plasma EV-borne laminin-332 in lymph node positive OSCC patients than in lymph node negative OSCC patients, which were both higher than healthy controls (*p* < 0.01). The protein load ex vivo fluorescent signals from the cervical lymph nodes of mice orthotopically implanted with PKH-26-labeled laminin γ2-deficient EVs (from LN1-1 LAMC2-knockdown cells) demonstrated a significant decrease in uptake relative to the corresponding controls (*p* < 0.01), suggesting a reduced ability to drain into lymph nodes in comparison with the control EVs [44].

Overall, these data indicate that in combination it may be that various salivary MV biomarkers including HSP90α, HSP90β, TRAP1, HSP105, VEGF-C, annexin V and laminin-332 could be of use in tumour detection, staging and prognosis.

### 2.4. Directed microRNA Cargo of OSCC-Derived EVs

Differential microRNA expression profiles of OSCC-EVs have been reported from both in-vitro and clinical studies. Rabinowits et al. (2017) compared the microRNA expression of tongue OSCC-derived EVs, matched benign tissue as well as plasma from one single patient [20]. They found significant differences in the microRNA expression profiles of tumour and matched benign tissue, with nine differentially upregulated and seven downregulated in tumour tissue out of 359 microRNAs found [20]. Comparison of the microRNA present in exosomes isolated from the conditioned media from 4 HNSCC cell lines: H413 (from buccal mucosa OSCC), Detroit 562 (pharyngeal cancer metastatic to pleura), FaDu (hypopharyngeal cancer) and Cal 27 (tongue OSCC), with similarly isolated EVs from normal gingival keratinocytes identified 32 common differentially abundant microRNA in the 4 HNSCC cell lines [56]. Further, comparison of the microRNA present in EVs isolated from the saliva of OSCC patients (*n* = 5) and 5 cancer-free controls found several of the microRNA that were upregulated in cancer culture cells were increased in salivary exosomes of HNSCC patients when compared to controls [56].

In a similar study comparing the concentration, size and microRNA content of EVs from the saliva of 5 OSCC patients and 5 healthy controls, Gai et al. (2018) showed that two microRNAs, miR-302b-3p and miR-517b-3p, were expressed only in OSCC patients while a further two, miR-512-3p and miR-412-3p, were present in greater abundance in comparison to EVs from the saliva from healthy controls [16].

Salivary exosomal miRNA microarray profiling by He et al. (2020) [18] of 4 OSCC patients and 4 healthy controls found 50 miRNA were significantly increased (fold change 3.60 to 345.62) and 59 miRNA were decreased (fold change 0.49 to 0.02). Particularly, miR-24-3p was significantly increased (fold change 121.54). The level of miR-24-3p was 5.73-fold higher in cancer patients than in healthy controls (*p* < 0.01) in a validation cohort of 45 OSCC vs. 10 healthy controls with an AUC of 0.738 (95% CI: 0.589–0.886, *p* = 0.02). The molecular mechanism of miR-24-3p in tumorigenesis was also investigated using plasmid transfection of HSC6 and SCC25 OSCC cell lines with the overexpression of miR-24-3p significantly increasing the colony formation and growth rate of OSCC cells. PER1 was recognized as a predicted target of miR-24-3p with its levels significantly decreased (*p* < 0.01) in OSCC patients compared to normal controls [18]. MicroRNA cargo of OSCC-derived EVs continue to be of interest as biomarkers and therapeutic targets.

### 2.5. Selective microRNA Cargo Loading into EVs in OC and Oral Dysplasia

Quantitative PCR profiling of EVs isolated from plasma of patients with OSCC showed that miR-21, miR-27b and miR-27a were specifically enriched in EVs compared to the non-EV fraction (*p* < 0.05) [21]. Comparing microRNA content of CAL27 cells vs. CAL27-derived EVs, demonstrated selected packaging with several microRNA differentially expressed between cells and EVs including miR-7d-5p, miR-130a-3p, miR-181a-5p, miR-107, miR-21-5p, miR-103a-3p, miR-22-3p and miR-20a-5p [21]. Dickman et al. (2017) [57] also demonstrated selective packaging of microRNAs into EV of OSCC and oral dysplasia cell lines, showing miR-142-3p, miR-451a, miR-150-5p and miR-223-3p were consistently dysregulated compared to their donor cells. Furthermore, miR-142-3p was found to be consistently increased in concentration in donor cells upon shRNA-mediated inhibition of Rab27A, which functions in a pathway responsible for selective packaging of microRNAs into small EVs [57]. Further evaluation of miR-142-3p, for its role in promoting cancer growth, found it to target TGFBR1, reducing its expression in donor cells leading to suppressed tumour growth and colony formation. Conversely, release of miR-142-3p in the small EVs was found to reduce TGFBR1 activity and promote tumour growth both in vitro and in vivo [57].

### 2.6. OSCC-Derived EVs Influence Phenotypic Change and Confer Tumorigenicity

The EV cargoes can influence expression and behaviour of target cells. Qadir et al. (2018) [32] measured mRNA transcripts following exposure of normal oral keratinocytes to exosomes isolated from 8 cells lines: 3 OSCC (Ca1, CaLH2, SQCC/Y1), one transformed malignant (SVFN8), one premalignant buccal oral keratinocyte (SVpgC2a) and 3 normal oral keratinocytes (OH113, NK4, NOK368). Exposure to exosomes from all cell lines impacted expression of mRNA transcripts involved in matrix modulation, cytoskeletal remodelling, immune response, lipid metabolism and membrane trafficking; whereas OSCC cell line-derived exosomes specifically modulated matrix and membrane remodelling, cell cycle, differentiation, apoptosis and transcription and translation [32].

Kawakubo et al. (2016) [28] found that purified exosome samples derived from cell culture supernatant of the metastatic cell line SQUU-B induced the invasive properties of non-metastatic cells (SQUU-A). This was associated with reduction in mRNA expression of *KRT13* [28] coding cytokeratin 13, the down-regulation of which is strongly linked to malignant transformation [58]. Sento et al. (2016) revealed that OSCC cell-derived exosomes promote tumour cell proliferation, migration and invasion of OSCC cells, potentially through the activation of Akt, ERK and JNK signalling pathways. Furthermore, OSCC-4 exosomes induced tumours of significantly larger size than parental OSCC-4 cells in a murine in vivo model [26]. Similarly, injection of EVs from the metastatic OSCC subline LN1-1 into mice induced tumours of increased volume than in mice injected the parental cell line OEC-M1 EVs or PBS (*p* < 0.01) [26].

Heparin can inhibit cancer cell exosomes internalization by blocking cell-surface heparin sulfate proteoglycan (HSPG) receptors [59,60]. Sento et al. 2016 [26], found that heparin treatment abrogated the uptake of OSCC-4 cell-derived exosomes by OSC-4 cells and subsequent effects such as promotion of cell proliferation, migration and invasion. However, these effects were transient in vitro and in vivo effects and required repeated heparin dosing [26]. Together, this data indicates that uptake of exosomes by OSCC cells uses similar mechanism(s) to that used by other cancer cell types and that exosomes can influence cancer cells in an autocrine and paracrine fashion.

The phenotypic changes induced by EVs are thought to be influenced by EV microRNA content. Sakha et al. (2016) [29] demonstrated that exosomes mediated transfer of microRNA to adjacent or distant cells for intercellular communication in OSCC cell lines when oncogenic microRNAs were delivered from a highly metastatic OC cell line HOC313-LM to the poorly metastatic cell line HOC313-P through exosomes. It was revealed that exosomal miR-342-3p and miR-1246 induced a pro-metastatic phenotype [29]. Involvement of exosomal delivered microRNA in metastasis was also demonstrated by Kawakubo-Yasukochi et al. (2018) [61]. The mRNA transcriptome of SQUU-A cells treated with exosomes from SQUU-A and SQUU-B had differential expression of 22 mRNA. Further analysis of 6 selected microRNAs revealed miR-200c-3p as a key pro-invasion factor that silenced the targets *CHD9* and *WRN*, significantly accelerating the invasive potential of SQUU-A cells [61].

Exosomes isolated from the murine HNSCC cell line SCCVII plus the human cell lines SCC90 (oropharyngeal SCC) and PCI-13 were injected into immunocompetent C57bL/6 mice with premalignant tumours induced using the 4NQO. The numbers of developing tumours per mouse increased in the SCCVII exosome treated group relative to the controls (6.2 vs. 3.2, *p* < 0.001) and per mouse tumour burden increased (*p <* 0.037) [62]. Importantly, the tumours had reduced inflammatory infiltrates. However, human cell line exosome injection did not result in significant tumour burden changes. Nonetheless, there was a significant reduction of immunofluorescence using both anti-CD4 and anti-CD8 antibodies in the murine tumours exposed to exosomes indicating there is a reduced inflammatory phenotypic effect induced by each EV type [62].

Further evidence of microRNA delivery by exosomes is shown by uptake of CAL 27-derived EVs by THP1 monocytes, with a corresponding increase in oncogenic miR-21-5p presence and associated activation of the NF-κB inflammatory pathway [21]. This was accompanied by an increase in measured levels of IL-6, CCL2, PEG2 and MMP9, suggesting a pro-inflammatory and pro-tumorigenic shift [21]. It was proposed that in particular, the transmission of miR-21 promotes monocyte migration and infiltration, which in turn promotes angiogenesis [21].

Further evidence of the importance of miR-21 in oral cancer was demonstrated by Li et al. (2019 [47], who using C3H mice showed that integrated anti-PD-L1 and miR-21 knockdown improved the antitumor effect of tumour-derived exosomes [47].

Collectively, these studies demonstrate that EVs derived from OSCC directly influence cellular phenotype by promoting cell proliferation, invasion and metastasis.

### 2.7. EV Mediated Fibroblast Interaction in OSCC

Exosomes derived from cultured human dermal fibroblasts (NHOK, NHDF) and oral keratinocytes (HaCaT) were shown to be produced at similar amounts but to possess different exosomal markers [39]. NHOK and NHDF exosomes were CD9, annexin V and Flotillin-1 positive, whereas HaCaT exosomes were only CD9 positive, pointing to altered protein packaging in these exosomes [39]. When co-cultured with healthy keratinocyte and fibroblasts, proliferation was suppressed, similar to the effect of the corticosteroid dexamethasone. Fibroblast exosomes had the more suppressive effect. Co-culture of these exosomes with OSCC cell line TR146 also suppressed proliferation at particular doses, but keratinocyte exosomes had the greater anti-proliferative effect [39]. Thus, non-malignant cells produce exosomes with differential effects on non-malignant and malignant cells.

Languino et al. (2016) [34] investigated whether exosomes purified from stromal fibroblasts are able to confer TGFβ signalling to SCC keratinocytes deficient in TGFβ ligand response. TGFβ signalling in cancer is complex and can have proliferative and anti-proliferative effects, dependent on the context. Languino et al. demonstrated that exosome transfer from TGFβ signalling-competent fibroblasts increased transforming growth factor-beta receptor II (TBRII) levels and the TBRII signal transducing protein SMAD2 phosphorylation in OSCC keratinocytes, while SMAD3 phosphorylation did not consistently change. Furthermore, stimulation of OSCC keratinocytes with recombinant TGFβ1 after exosome transfer resulted in a small, but further increase of phosphorylated SMAD2 [34]. Thus, exosome delivery and uptake influence TGFβ signalling.

Principe et al. (2018) [35] had a main focus to identify cancer-associated fibroblast (CAF)-enriched secreted molecules that are shuttled between fibroblasts and cancer cells and that can affect cancer cell progression. To this end, they have determined a comprehensive CAF proteome. They found that CAF secreted exosomes significantly increased SCC25 cell proliferation and migration compared with exosome-depleted media or controls. In addition, when OTSCC cells were treated with human recombinant microfibrillar-associated protein 5 (MFAP5), which they identified as enriched in the CAF secretome, cell proliferation and migration via activation of MAPK and AKT oncogenic pathways were observed [35]. Investigation of MFAP5 levels in patient tumours showed elevated levels in cancer-associated stroma [35].

In their analysis, Li et al. (2018) [36] observed that the expression of miR-34a-5p was significantly reduced in CAF- and SCC cell line (SCC25 and Cal-27)– derived exosomes. They demonstrated transfer of cy3-tagged miR-34a-5p to the OSCC cells. In xenograft experiments, miR-34a-5p overexpression in CAFs could inhibit the tumorigenesis of OSCC cells [36]. Sun et al. (2019) [38] observed that exosomes derived from CAFs and normal fibroblast (NF) medium when added to a medium of OSCC Cal-27 cells, exerted strong effects on upregulating MMP-3, MMP-9, N-cadherin and β-catenin. Furthermore, CAL-27 migration and invasion assays significantly increased when exposed to CAF exosomes (*p <* 0.05) [38]. The onco-microRNA miR-382-5p is associated with several cancers and has been shown to be more abundant in CAFs than in NFs [63]. Direct transfection of CAL-27 with miR-382-5p mimics promoted migration and invasion capacity [63].

OSCC-derived MVs may influence normal fibroblasts that in turn can influence tumour progression. The glycometabolic influence of MVs derived from CAL-27 OSCC cells was studied by Jiang et al. (2019) in HGFs (human gingival fibroblasts) from healthy individuals, PNFs (paracancerous normal fibroblasts) and CAFs 3 OSCC patients [23]. HGFs incubated with the OSCC-derived MVs internalized these tumour MVs (TMVs) and subsequently increased levels of CAF markers (FAP and Tn-c). TMV-treated HGFs also showed increased glucose and lactate production with an increased expression of GLUT1, PDK1 and MCT4, but a decrease in CaV1. Knockdown experiments on HGFs showed CAV1 knockdown caused increased expression of PDK1 and MCT4, but not GLUT1, indicating Cav 1 is related to aerobic glycolysis of HGFs through ERK1/2 activation pathway. Injection of a combination of untreated and TMV-treated HCFs with CAL-27 xenograft into nude mice found that treating with TMVs was associated with a significant increase in tumour weight (*p <* 0.05). The invasive potential of OSCC cells was demonstrated to be increased after being co-cultured with TMV-treated HCGs and partially reversed by knockdown of MCT1 in CaL-27 cells [23].

Effects of EVs derived from 5 primary CAF lines and 5 primary NF lines were studied on 4 TOSCC cell lines: HSC3 and SAS (higher invasive and metastatic potential) and the less aggressive SSC-15 and SSC-25 cell lines [37]. The invasive potential of HSC-3, SAS and SCC-15, but not SCC-25, was significantly increased (*p <* 0.05) by co-culture with individual CAF-EVs compared to NF-EVs and controls. HSC-3 was the most responsive to pooled CAF-EVs and invaded deeper (*p <* 0.01) and with smaller tumour islands (*p <* 0.05) when compared to NF-EVs and controls. Further, migration (*p <* 0.05) and apoptosis rate (*p <* 0.05) were increased. Microarray was performed on total RNA isolated from HSC3 cell line treated with CAF-EV or NF-EV, identifying thirty-two differentially expressed (FC ≥ 1.3) genes. These findings support CAF-EV mediated influence on OSCC behaviour and gene expression which may be those OSCCs that already possess higher invasive and metastatic potential [37].

Ding et al. (2018) [33] studied long non-coding RNA profiles in the transformation of NFs into CAFs within the OSCC microenvironment, particularly identifying upregulation of lncRNA LOC400211, also known as Lnc-CAF. Lnc-CAF is located in 14q23.1-q23.2, identified as a H&N SCC cancer risk locus [64]. Expression of Lnc-CAF in OSCC cell line HSC3-derived exosomes was found to be high. Incubation with HSC3 exosomes lead to uptake by NFs with resulting upregulation of expression of Lnc-CAF (*p* < 0.05) [33]. This is the only study to date reporting expression of lncRNA in exosomes in OSCC [33].

Together, these studies demonstrate a bi-direction EV mediated CAF influence in OSCC progression.

### 2.8. OSCC-Derived EVs Stimulate Changes in Endothelial Cells Consistent with Angiogenesis and Lymphangiogenesis

Several studies demonstrated effects from OSCC-derived EVs on vascular and lymphatic endothelial cell lines with changes in gene expression and protein abundances consistent with angiogenic and lymphangiogenic processes. Morioka et al. (2016) exposed HUVECs (human umbilical vein endothelial cells) and HDLECs (human dermal lymphatic endothelial cells) to exosomes isolated from 2 OSCC cells lines developed from tumours isolated from the same patient, one non-metastatic (SQUU-A) and one metastatic (SQUU-B). The HDLEC response to SQUU-A exosome exposure was an increase in VEGFR1, VEGFR2 and VEGFR3, whereas the response to exosomes from SQUU-B cells led to an increase in all VEGFRs, as well as VEGF-A, VEGF-C and VEGF-D. HUVEC response to exposure to exosomes from each OSCC cell lines was an increased level of mRNA and protein of only VEFGR2 [40]. Exposure to exosomes from SQUU-B cell lines lead to increased branching of HDLEC, indicating stronger tube formation, but neither exosome population affected HUVEC branching [40]. In contrast, de Andrade et al. (2017) [42] have shown OSCC cell line SSC15 derived EVs induced significant HUVEC tube formation, migration and increased apoptotic bodies in comparison to HSC3 cell line EV that significantly inhibited tube formation and proliferation [42]. SCC15 and HSC3 release EV similar in size and concentration, but HSC3 cells exhibit higher CD63 and annexin II levels. Thus, pro and anti-angiogenic effects can be exerted on HUVEC by EVs derived from different cell lines. Whether CD63 and annexin II exposure influences HUVECs remains to be determined.

Aberrant activation of the sonic hedgehog (Shh) pathway in OSCC specimens was shown to be closely related to angiogenesis via the RhoA/ROCK signalling pathway [41]. Using immunohistochemistry to detect sonic hedgehog (SHH) in primary and metastatic FFPE specimens of 80 OSCC, Huaitong et al. (2017) [41] found that SHH was positively associated with micro-vessel density, TNM stage, tumour recurrence and lymph node metastasis. SHH was assessed by Western blot and shown to be abundant in the OSCC cell line CAL 27; however, the amount of SHH in CAL 27-derived microvesicles was up to 5-fold that of the cell line itself. HUVECS treated with CAL 27-derived microvesicles followed by Matrigel culturing were found to increase tube formation. Furthermore, this effect was blocked utilizing exoenzyme C3 transferase and shRNA targeting RhoA. This together supported that OSCC-derived SHH in microvesicles may modulate angiogenesis and vascular bed preparation of primary tumours and future metastatic sites [41]. Therefore, it appears that cytoskeletal system can enhance cancer cell invasiveness and the metastatic potential via exosomes.

Ludwig et al. (2018) [43] aimed to analyse to what extent exosomes produced by OSCC cell lines, the human papilloma virus-(HPV)-negative line PCI-13 and HPV-positive lateral tongue SCC cell line UMSCC47 play in blood vessel formation in vitro using HUVEC and an immunocompetent orthotopic mouse model. Culturing the tumour cells under hypoxic conditions significantly increased the yield of total exosome proteins for both cell lines. Co-incubation of HUVECs for 24 h with 20 mg of PCI-13–derived exosomes caused an increase in *VEGF* mRNA levels and *IGFBP-3* mRNA expression levels in the recipient cells (*p <* 0.05), suggesting growth promotion. However, no significant changes were seen after co-incubation of HUVECs with UMSCC47-derived exosomes. Nor were significant differences observed in internalization of PCI-13 and UMSCC47-derived exosomes by HUVECs. Results using the in vivo 4-NQO oral carcinogenesis model [65] showed tongue base cell line UMSCC90, PCI-13 or murine cell line SCCVII-derived exosome-treated groups have an increased amount of vascular structures within the tumour tissue. Overall, the tumour-derived exosomes promoted angiogenesis [43]. Plasma analysis showed that relative to healthy controls, patients with HNSCC with active disease have elevated levels of exosomes that interact with HUVECs and stimulate their proliferation [43]. Wang et al. (2019) [44] found that incubation of human dermal lymphatic endothelial cells (LEC) with metastatic subline LN1-1- EVs did not affect growth but significantly increased migration and tube formation of LEC when compared to LEC incubation with non-metastatic parent cell line OEC-M1-derived EVs [44].

Thus, pro- and anti-angiogenic effects can be exerted on HUVEC and other non-malignant cell lines by EVs derived from different cell lines. In addition, specific tropism shown by the HUVEC and HDLEC cells [40] points to avenues of investigation regarding the metastatic process.

### 2.9. Immune Influence of OSCC Derived EVs

Correlation has been found between Fas ligand (FasL) expression on tumour cells and T cells undergoing apoptosis within the tumour microenvironment in subsets of OSCC patients [66,67]. Kim et al. (2005) were able to isolate MVs from the sera of 27 OSCC patients, whereas they were unable to isolate MVs from healthy controls. They found that 21 patients had Fas ligand positive MVs (FasL+) and were able to induce apoptosis of Jurkat and T-cell blasts. The biological activity of the FasL+ MVs was partially blocked by ZB4 anti-Fas monoclonal antibody. In addition, FasL levels were found to be correlated with the patient tumour stage, highlighting that FasL+ MVs may play a role in tumour bed immune response [12].

The effects of EVs from tongue OSCC cells on the phenotype and cytotoxic activity of immune cells in vitro and on the innate immune system in vivo using a zebrafish model was assessed by Al -Samadi et al. (2017) [68]. They demonstrated that activated immune cells significantly decreased the proliferation and invasive abilities of tongue OSCC cell lines. Furthermore, EVs from SCC-25 increased the cytotoxic activity of CD8+T and NK cells more than those from HSC-3 cells. In zebrafish, the amount of IL-13 mRNA was decreased following exposure to SCC-25 derived EVs [68], suggestive of reduced B-cell and macrophage activation, amongst other possible effects. Wang et al. (2018) [69] showed that OC-derived exosomes directly enhanced the cytotoxicity of NK cells. In addition, NF-κB-activating kinase-associated protein 1 (NAP1) was significantly enriched in the corresponding donor cells. Following internalization of NAP1 derived from OSCC cells, there was an augmentation of levels of downstream molecules associated with NK cell activation and tumour-suppressive function [69]. Xiao et al. (2018) [46] demonstrated that conditioned media from OSCC-exosome-activated macrophages significantly promoted the migration of OSCC cells. However, this was not found with exosomes derived from normal keratinocytes and leukoplakia cell lines [46].

OSCC-stromal mononuclear cells or macrophages can differentiate into subtypes, mainly M1 with antitumor effects, M2 with tumour-promoting effects and with M2 subtype macrophage infiltration levels negatively correlated with prognosis in OSCC [70]. Exosomes from OSCC cell lines SSC-9 and CAL-27 were cultured by Cai et al. (2019) [48] with monocytic cell line THP-1- derived macrophages to study exosome effect on polarization of macrophages to M2 subtype. These co-cultured macrophages showed higher expression levels of protein markers of M2 macrophage subtype: CD163, CD206, Arg-1 and IL-10. Additionally, the medium of the coculture promoted the proliferation and invasion of the source OSCC cell lines. OSCC tissues were found to have high expression miR-29a-3p, low suppressor of cytokine signaling 1 (SOCS1) and highly expressed phosphorylated signal transduction and transcriptional activator 6 (p-STAT6) compared to adjacent tissue [48]. Inhibition of miR-29a-3p in the OSCC cell lines found that co-culture with subsequent derived exosomes led to inhibition of M2 phenotype conversion, higher levels of SOCS1 and lower levels of STAT6 expression. Accompanying the invasive and proliferative ability following placement of the culture medium with the sources OSCC cell line was also found to be decreased [48]. This demonstrated an EV-mediated interaction of tumour promotive effects through promotion of M2 macrophage differentiation, mediated by EV-transferred miR-20a-3p via the SOCS1/STAT6 signalling pathways.

Together the data indicate that EVs carry chemical signals that direct the immune system to reduce function and also increase pathogenic potential of malignant cells.

### 2.10. Modulating OSCC-EV Production and Packaging

There has been some evidence presented that extracellular factors impact EV production and contents. Annexin A1 (ANXA1) has been implicated as an important factor in HNSCC cellular proliferation via EGFR signalling pathway [71]. ANXA1 has been negatively correlated with increasing tumour grade in OSCC [72,73]. Raulf et al. (2018) [71] observed an inverse relationship between ANXA1 levels and EGFR, with dysplastic tissue rich in EGFR and poor in ANXA1. Down-regulation of ANXA1 resulted in increased EGFR activity and downstream PI3K-AKT signalling that may be via exosomal EGFR. It was also shown that shRNA knockdown of *ANXA1* in HN5 tongue OSCC cell line resulted in reduced production levels of EVs and concurrent reduced levels of exosomal phosphorylated-EGFR [71]. Furthermore, functional studies using shRNA to knockdown ANXA1 resulted in a clear increase in cellular proliferation in 4 different HNSCC cell lines.

When Momen-Heravi et al. (2019) challenged cultured CAL-27 OSCC cells with different doses of LPS and ethanol, it resulted in increased EV production with particular increase in larger subset of microvesicles compared to the smaller exosomes, although this did not appear to markedly alter microRNA cargo of EVs [21].

Normoxic and hypoxic conditions were shown to affect packaging of tumour-derived exosomes and downstream T cell activity. Li et al. (2016) [27] found that exosomes derived from hypoxic OSCC cells increased the migration and invasion of OSCC cells in a HIF1α and HIF-2α–dependent manner. Out of 108 microRNAs differentially expressed under hypoxic conditions, miR-21 was the most significantly upregulated. Notably, miR-21 depletion in hypoxic OSCC cells led to decreased miR-21 levels in exosomes and significantly reduced cell migration and invasion. This same group later demonstrated that tumour derived exosomes from Cal-27 and SCC-9 cells produced under normoxic conditions activate cytotoxicity of γδ T cells against these same OC cell lines, an effect attenuated under hypoxic conditions. Similarly, C3H mice injected with SCCVII and previously immunized with normoxic exosomes, had significantly decreased xenograft tumour growth, whereas this was significantly increased by hypoxic exosomes. It was found that myeloid-derived suppressor cells (MDSCs) played an important role in hypoxic exosomes EV-induced suppression of γδ T-cell function, purported to be mediated by a miR-21/PTEN/PD-L1 regulation axis [27].

OSCC-EV production appears to be influenced by tumour bed environment and may in turn have downstream influence on tumour behaviour.

### 2.11. Therapeutic Influence on OSCC-EVs

Melatonin has been demonstrated to reduce OSCC tumorigenesis in vivo [74]. Hunsaker et al. (2019) [75] studied the effect of the addition of melatonin to culture on exosomal expression of the OSCC-associated microRNAs, miR-21 miR-133a and miR-155, in the OSCC cell lines SSC25, CAL27 and SSC9. Increased expression of both miR-21 [76] and miR-155 [76] have been associated with poor prognosis, whereas miR-133-3p has been reported to inhibit OSCC in vitro [77]. Exosomal miRNA expression assessed by qPCR following addition of melatonin found significantly increased expression of miR-21 from all three cell lines (*p <* 0.05), significant reduction of miR-155 expression (*p* < 0.05), but no impact on miR-133a expression [75]. This exosomal microRNA modulation did not necessarily clarify the role of melatonin’s possible affects against oral cancer but indicates the potential that therapeutic regimes could have on OSCC-EVs.

### 2.12. OSCC-EV Modulated Influence on Therapeutic Response

Selective EV packaging and production by OSCC cells appears to influence resistance to chemotherapeutic agents, such as the nucleotide binding, cisplatin, and human-mouse chimeric monoclonal antibody, cetuximab, which binds to EGFR. Liu et al. (2017) [78] found that miR-21 was highly expressed in exosomes derived from two cisplatin-resistant OSCC cell lines, SCC-9-R and HSC-3-R, which were developed from SCC-9 and HSC-3 cells, respectively. Treatment with EVs derived from cisplatin-resistant OSCC cells induced cisplatin resistance in the parental cells and was associated with EV transference of miR-21. Similar findings were demonstrated in a subcutaneous mouse xenograft model where exosomes derived from HSC-3-R and HSC-3 parent cells were injected together with cisplatin [78]. Other investigators showed that HSC-3 cells abundantly express EGFR, secreted with EVs upon EGF stimulation [31]. The OSCC EGFR-containing EVs had the ability to enter and transform human oral squamous epithelial cells (RT7) to initiate epithelial-mesenchymal transition, effects of which were largely blocked by the anti-EGFR antibody cetuximab. These EV effects were enhanced by EGF priming suggesting that EGFR signalling could affect EV properties [31]. EGF stimulation was also shown to promote the progression to mesenchymal traits in OSCC cells, which was reduced by cetuximab [31]. Cetuximab promoted secretion of EGFR-EVs by OSCC cells and failed to inhibit EGF-driven secretion of EGFR-EVs. Cetuximab was also found to be robustly secreted with the EGFR-EVs by the OSCC cells, which may be a novel mechanism of action for a drug [79].

EVs were isolated from de novo (H314) and adaptive (H103/cisD2) cisplatin-resistant OSCC cell lines and cisplatin-sensitive OSCC cell line (H103) [80]. Protein quantification showed that resistant cell lines produced 2 to 2.7-fold more EV protein per million cells than H103 cells. H103 cells showed the highest level of EV protein markers (CD9, CD63, CD81) and heat shock protein HSC70. Compared to H103, EVs derived from H103/cisD2 demonstrated 104 differentially expressed proteins and H314 demonstrated 161 differentially expressed proteins. In contrast, H314 differed in only 4 proteins from comparison to H103/cisD2. The proteins expressed in resistant cell lines belong to PACSIN3, mainly functioning in vesicle mediated transport, along with proteins responsible for sodium-potassium ion transportation. When treated with cisplatin, H103 cells released 4 times more EVs, but still significantly less than the resistant cell lines. It was found that less cisplatin accumulated in the resistant cells, but higher levels were found with derived EVs, demonstrating efflux. Further, inhibiting EV secretion using Lansoprazole resulted in a decreased viability following cisplastin administration of resistant H314 cells (*p <* 0.05), but no significant difference in H103 or H103/cisD2 viability [80].

Thus, EVs play a role in OSCC resistance as a drug efflux mechanism that may be an area for future therapeutic development.

### 2.13. EVs for Therapeutic Delivery in OSCC

EVs have been highlighted as an interfering factor of drug action, but they have also been shown to be potential carriers for targeted drug delivery in OSCC. Yu et al. (2017) [81] described traceable targeted therapy, also known as theragnostics, embedding circulating microparticles (CMPs) purified from peripheral blood and saliva of OSCC patients with ultra-small near-infrared-fluorescent magnetic quantum dots (Ag2Se@Mn QDs) for tracking, labelling and subsequently injection into mice. They discovered that the CMPs remain stable in circulation for at least 48 hours and showed natural targeting and accumulation at grafted tumour sites, without demonstrating tumour promoting effects. Further, they were able to embed an anti-tumour small interfering (siRNA) into the CMPs, essentially producing therapeutic nano-vectors that lead to suppression of the tumour xenograft growth (*p <* 0.01) [81].

Hoornstra et al. (2018) [52] examined the therapeutic use of EVs to increase the bioavailability of curcumin, the bioactive component of turmeric, known for its anti-carcinogenic properties [82]. They loaded curcumin into *Candida galbrata* EVs and added them to the aggressive HSC-3 cell line and less aggressive SCC-25 OTSCC cell line for comparison with unloaded curcumin. The EV curcumin loading improved bioavailability, but the anti-carcinogenic effect on OTSCC cells did not increase [83].

*COL10A1* is a gene found to be down-regulated in expression in most normal tissues and increased in expression in multiple cancer types [84]. Following demonstration that *COL10A1* was upregulated and miR-101-3p down-regulated in OSCC tissues and cell lines, Xie et al. (2019) [50] studied the effect of primary human bone marrow mesenchymal stem cells (hBMSCs) transfected with miR-101-3p-Cy3-derived exosomes on the TSCC cell line, TCA 8113. Using differential fluorescence, they demonstrated that miR-101-3p was transferred from donor hBMSC to receptor TCA8113 cells and this exosomal miR-101-3p downregulated *COL10A,* an effect prevented by suppressing exosome secretion using GW4869 and DMA. Further, the hBMSC-derived exosomal transfer of miR-101-3 significantly repressed cell invasion and migration (*p* < 0.05) and reduced the colony-forming ability of TCA8113 cells (*p* < 0.05) [50]. Continuing onto a further in vivo model of nude mice injected with 1 × 10^7^ TCA8113 cells resuspended with 200 μL PBS, then treated with subcutaneous injection of exosomes with hBMSCs-miR-101-3p, showed reduced tumour volume and weight compared to those injected with hBMSCs-miR-NC and PBS [50], confirming a function for miR101-3p in regulating cancer. *COL10A1* is a gene found to be down-regulated in expression in most normal tissues and increased in expression in multiple cancer types [84]. Following demonstration that *COL10A1* was upregulated and miR-101-3p down-regulated in OSCC tissues and cell lines compared to controls, Xie et al. (2019) [50] studied the effect of primary human bone marrow mesenchymal stem cells (hBMSCs) transfected with miR-101-3p-Cy3-derived exosomes on TSCC cell line TCA 8113. Using differential fluorescence, they demonstrated that miR-101-3p was transferred from donor hBMSC (human bone marrow stromal cells) to receptor TCA8113 cells and this exosomal miR-101-3p downregulated *COL10A,* whereas suppressing exosome secretion using GW4869 and DMA prevented this effect. Further, the hBMSC-derived exosomal transfer of miR-101-3 significantly repressed cell invasion and migration (*p <* 0.05) and reduced the colony-forming ability of TCA8113 cells (*p <* 0.05) [50]. Continuing onto an in vivo model, nude mice injected with 1 × 10^7^ TCA8113 cells and then treated with subcutaneous injection of exosomes with hBMSCs-miR-101-3p had reduced the tumour volume and weight compared to controls [50], confirming a function for miR101-3p in regulating cancer.

MicroRNA miR-138 was suggested to function as a tumour suppressor and has been shown to target CTLA-4 and programmed cell death 1 (PD-1) in CD4+T cells [85]. Li et al. (2019) [86] utilised lenti-miR-138 virus γδT cell-derived extracellular vesicles (γδTDEs) as a drug delivery system in the treatment of OSCC. Moreover, delivery of miR-138 with γδTDEs had synergistic inhibition on CAL 27 cells, both in vitro and in nude mice, in vivo. Pre-immunization using miR-138-rich γδTDE inhibited growth of OSCC (SCCVII cell) tumours in immunocompetent C3H mice, but not in T-cell lacking nude mice. The γδTDE with miR-138 increased CD8+T cell proliferation, IFN-gamma production and cytotoxicity (at 10:1 or greater) against OSCC cells. γδTDEs delivered miR-138 significantly decreased the CD8+T cell expression of l PD-1 and CTLA-4 at both mRNA and protein levels (*p* < 0.05) [86]. MicroRNA miR-138 was suggested to function as a tumour suppressor and demonstrated to target CTLA-4 and programmed cell death 1 (PD-1) in CD4+T cells [85]. Li et al. (2019) [86] utilised lenti-miR-138 virus γδT cell-derived extracellular vesicles (γδTDEs) as a drug delivery system in the treatment of OSCC. Moreover, delivery of miR-138 with γδTDEs had synergistic inhibition on CAL 27 cells both in vitro and in nude mice in vivo. Pre-immunization using miR-138-rich γδTDE inhibited growth of OSCC (SCCVII cell) tumours in immunocompetent C3H mice, but not in T-cell lacking nude mice. The γδTDE with miR-138 additively increased CD8+T cell proliferation, IFN-gamma production and cytotoxicity (at 10:1 or greater) against OSCC cells. γδTDEs delivered miR-138 significantly decreased the CD8+T cell expression of l PD-1 and CTLA-4 at both mRNA and protein levels (*p <* 0.05) [86].

UCH-L1 is a multifunctional protein with de-ubiquitinating (DUB) activity suggested to play a major pro-metastatic role in certain carcinomas with involvement in secretion of EVs [87,88]. Kobayashi et al. (2019) [89] treated carcinoma cells with a small molecule inhibitor of UCH-L1-DUB activity, LDN-57444 in both free and incorporated into polyoxazolinemicelles (LDN-Pox) forms. Both forms reduced the invasive invasiveness of OSCC line HSC-3 cells (*p <* 0.05) [89].

These studies clearly demonstrate that EVs have the capacity to enhance the delivery of therapeutic agents and in turn have the potential to provide targeted cancer therapy.

### 2.14. EVs and OPMDs

#### 2.14.1. Oral Leukoplakia and Dysplasia

Dickman et al. (2017) using a qPCR microarray assessing 742 miRNAs, found that the microRNA of small EV cargo from an oral dysplasia cell line (DOK) was more analogous to the other OSCC cell lines than to normal keratinocytes [57]. Qadir et al. (2018) studied the impact of EVs derived from premalignant buccal oral keratinocyte (SVpgC2a) and malignant-transformed sub line of SVpgC2a and SVFN8 upon the SVpgC2a cell line itself. Transfection with SVFN8 exosomes triggered morphological change resembling senescence; however, there was no evidence of senescence associated β-galactosidase activity, nor significant mRNA modulation of senescence/apoptotic genes, suggesting that the exosomes modulated but did not directly activate differentiation [32].

Li et al. (2019) [49] derived exosomes from cultured primary mesenchymal stem cells (MSCs) from normal oral mucosa (N) (*n* = 3), oral leukoplakia with dysplasia (LK) (*n* = 3) and oral carcinoma (Ca) (*n* = 3) and studied their effect on cell lines SCC15 (OSCC cell line) and DOK. Exosomes derived from pre-malignant LK-MSCs (LK-exo) as well as Ca-MSCs (Ca-exo) accelerated the proliferation, invasion and migration of DOK and SCC15 cells, compared with the exosomes derived from the N-MSCs (N-exo) (*p <* 0.01). This enhancing effect was also demonstrated in a TGF-β1-3D coculture model of MSCs and SCC15, cells and inhibited with the utility of exosomal secretion inhibitor GW4869 [49].

Microarray analysis demonstrated that expression levels of miR-4433a and miR-8485 differed between pooled N-exo, LK-exo and Ca-exo. Both DOK cells and SCC15 treated with LK-exo and Ca-exo exhibited elevated expression of miR-8485 (*p <* 0.05). Additionally, miR-8485 mimics caused rapid growth (*p <* 0.05) and increased invasion ability (*p <* 0.05) of both DOK and SCC15 cells and an miR-8485 inhibitor led to the inhibition of these processes [49]. It would appear that EVs derived from OPMD are comparable to those derived from OSCC cells, including tumorigenic capabilities.

#### 2.14.2. Oral Lichen Planus

Differential expression in patients with OLP of both salivary and plasma derived exosomes has been reported. Byun et al. (2015) compared the salivary exosomal microRNA profiles of 16 OLP patients with 8 healthy controls using miRNA microarray analysis and TaqMan quantitative PCR and concluded that miR-4484 was significantly upregulated in the OLP patients, highlighting its potential use as a future prognostic and diagnostic marker of OLP [24]. Peng et al. (2018) compared microarray analysis of pooled plasma derived exosomal microRNA from 19 OLP patients with 11 controls and found significant (fold changes >2 or < 0.5 and *p <* 0.05) increased expression of miR-34a-5p, miR-130b-3p and miR-29c-3p and decreased miR-301b- 3p and miR-144-3p. This group presented the first report comparing clinical disease severity with exosomal findings. They correlated increasing expression of exosomal miR-34a-5p with an increasing reticular, atrophic and erosive (RAE) severity score [25]. They further highlighted the particular impact of exosomes derived from patients with erosive type mucosal disease on T-cells. Plasma-derived exosomes from 12 patients with OLP (6 erosive and 6 non-erosive forms) were PKH67-labelled and co-incubated with Jurkat (T lymphocyte) cells. Significant internalization of OLP-exosomes by T-cells occurred within 48 h (*p <* 0.05). Although this uptake did not result in significant T-cell activation, T-cell proliferation was significantly increased with incubation with erosive LP-derived exosomes, but was not observed in non-erosive LP-derived exosomes or controls. Incubation with erosive LP-derived exosomes significantly (*p <* 0.05) promoted T-cell migration and suppressed relative to control EV incubation [51]. Unique exosomal microRNA profiles identified from patient samples could be of diagnostic and prognostic value in OLP; however, this requires significant further investigation.

## 3. Discussion

Overall, interest and research regarding mechanisms and roles of EVs in OSCC and OPMD has profoundly expanded in the last five years. EVs are an integral part of cell-to-cell communication networks. The oral cavity could potentially provide a unique and useful direct access to the tumour microenvironment that may allow for an opportunity to sample and interpret the cell-to-cell communications within the tumour environment. In this way, the study of OC-associated EVs could present an important model for other solid tumours.

There has been a consistent recent description of change in the quantitative and qualitative production of EVs in OSCC with further demonstration that selective packaging occurs by OSCC relative to tumour aggressiveness of the parent cell as well as local tumour environment factors. Larger tumour-derived vesicles have been reported in human circulation in prostate cancer and identified to carry the majority of tumour derived DNA, reflecting the genomic aberration of the tumour of origin [90]. Characterisation between exosomes and microvesicles remains challenging, particularly when considering OSCC-associated increase in vesicle size. Interestingly, Zhong et al. (2019) found research on microvesicles alone did not note a size increase in EVs in OSCC [19].

Morphological and molecular differences in oral fluid-derived EVs from OSCC patients may reflect an environment in direct contact with tumour tissues, while findings from circulating EVs support that tumour related differences are not just localized. Salivary and oral fluids likely include EVs sourced from different cells types and may give insight into the local tumour microenvironment. The difference highlighted in morphology, molecular surface markers, proteins, transcriptomic and small RNA cargo may be further integrated as composite biomarkers to identify the presence of OSCC early, but may also indicate metastatic behaviour [14,53] and potentially guide therapy.

The majority of clinical samples utilized in the study of EVs in OSCC were salivary or oral fluid-derived samples. It would be of particular interest to compare and correlate salivary findings with other cancer types. Reports are beginning to include comparisons of plasma, serum and salivary EV findings in OSCC, and such comparisons would also be useful in OPMDs. Dysregulated epithelial growth and immune interaction observed in OPMDs present important models for the understanding of complex gastrointestinal and aerodigestive mucosal carcinogenesis that may not be as easily observable in other sites. What is learnt in oral premalignancy may be critical to understanding precursors in other solid malignancies.

OSCC-derived EVs’ ability to exert both pro- and anti-angiogenic effect on endothelial cells of venous and lymphatic origin appear to be cell-line and recipient cell specific. Particularly, OSCC-EV derived influence on recipient cell VEGF [40,43] and Shh via RhoA/ROCK [41] signalling pathways has been demonstrated. OSCC-EV cargo influence on angiogenesis may not predictably parallel clonal aggressiveness with EVs derived from the highly metastatic cell line (SQUU-B) that is associated with stronger endothelial cell tube formation, when compared to a non-metastatic line (SQUU-A) from the same patient [40]. However, in contrast, a cell line with a highly invasive behaviour (HSC3) was found to have significantly inhibited tubulogenesis and endothelial cell proliferation relative to a cell line with low invasive behaviour (SCC15) [42].

EV influence in the OSCC tumour microenvironment is not sourced from carcinoma cells alone. It has been clearly demonstrated that cell-to-cell EV influence is between pre-malignant cells and normal keratinocytes [32], as well as CAFS, macrophages and immune cells that have been demonstrated to promote phenotypic changes of OSCC cells, including cell proliferation, migration and invasion [35,46]. Two studies supported the priming influence upon innate NK cell response [45,68], although it was interesting to observe that cytotoxic activity of CD8+T and NK cells was increased further by a less invasive cell line (SCC-25) compared to a highly invasive cell like (HSC-3) [68].

OSCC EVs appear to be a mechanism by which immune response can be modulated both near and distant to the oral cavity. Circulating pro-T-cell apoptotic FasL+ve microvesicles found in the majority of OSCC patients’ sera have been shown to correlate with tumour burden and disease spread [12].

Dickman’s methodology of targeting exosome export protein Rab27A is interesting and potentially resulting in a paradigm shift, as the resultant feedback loop causes a reduction in malignant features [57]. In parallel, knockdown of ANXA1, thought to play a tumour-suppressive role in regulating exosomal release of tongue OSCC cell line HN5, led to reduced secretion of EVs in the exosome range and labelled exosome associated phosphorylated EGFT expression [71]. These in vitro findings may have relevance to the two mechanisms of anti-EGFR cetuximab highlighted: transference of microRNA-21 from cetuximab-resistant OSCC [78]; as well as robust secretion of EGFR-EVs by OSCC cells [79]. Developing therapeutics that modify mechanisms involved in exosome export may present avenues to augment current treatments.

Packaging EVs with targeted molecules presents an exciting possibility for future precision medicine in OSCC. Study of individual tumours may allow for target design using a patient’s own blood cells to define package-targeted therapy. EVs in the field of theragnostics, which combines the use of diagnostic imaging modalities with targeted therapy, may make such an approach an observable treatment delivery [81]. The therapeutic benefits of EV-loading for chemotherapeutic targeting may not necessarily increase the efficacy of the therapy [83]. However, targeted delivery and uptake with visualization would appear to allow increased precision and reduction in the effects to bystander tissue.

In conclusion, EV research in OSCC has been recently expanding on several fronts. What appears to be a trend, particularly across in-vitro studies, are the influence of OSCC-derived EVs. They are largely clone dependent and caution in translation needs to be considered, given the limited pool of OSCC cell lines available for investigation. There is a paucity of studies that isolate and identify EVs sourced from individuals with potentially malignant oral mucosal disorders as well as in vitro and in vivo models. It may be that studies are reporting EV cargo related findings without including the necessary EV isolation and characterisation steps in their methodology. Knowledge of the influence of OSCC-derived EVs upon OPMD models present important clinical relevance. In order to shed further insight into EV cargo, studies in OSCC and OPMDs should include an EV characterization step.

## 4. Materials and Methods

This systematic review was updated to December 17, 2019. Results are reported according to the Preferred Reporting Items for Systematic Reviews and Meta-Analyses (PRISMA) statement [91]. The search strategies according to the syntaxes of each database are displayed in Table S1.

### 4.1. Selection Criteria

No limits were placed on the search and articles reported in non-English languages were excluded. All papers including case reports and conference abstracts were included. No restriction was placed on the date of publication. Both animal and in vitro studies were included. Withdrawn/retracted studies, review articles, commentaries, opinion articles, letters to the editor and unpublished articles were excluded. Studies were included if they discussed either OSCC or OPMDS and their subcategories were addressed in addition to EVs being isolated and identified in their methodology.

### 4.2. Study Selection

**Figure 2:**
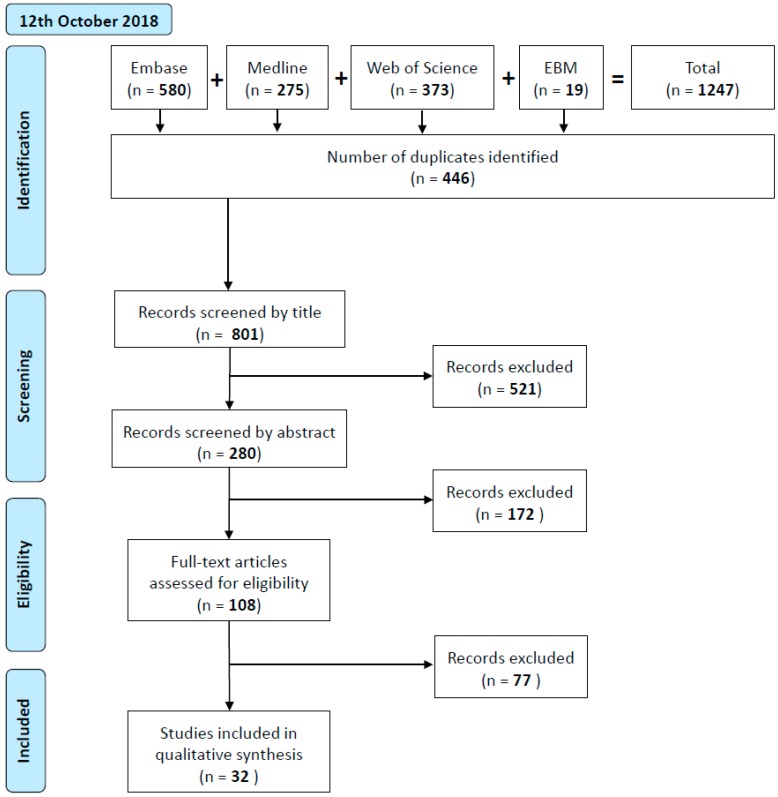
Flow diagram of selection of studies eligible for inclusion.

The selection of studies eligible for inclusion in this review was conducted by following the five steps as illustrated in Figure 2.

Step 1: Electronic literature searches were conducted in the PubMed (Ovid), Embase (Ovid), Evidence Based Medicine (EBM) Reviews (Ovid) and Web of Science (ISI) databases on 12 October 2018. The citations identified for further evaluation were imported into the reference management software package Endnote X8 (Clarivate Analytics, Philadelphia, PA, USA). De-duplication was achieved by the Endnote software and manually by two reviewers (TY, AC). Step 2: Ineligible records were excluded based on sequential review of title only (TY, AC). Each subsequent step was conducted independently by two reviewers (TY, NP) separately and blinded to each other. Step 3: Titles and abstract were reviewed, and Step 4: Full text review was undertaken. At each step, the reviewers’ decisions were compared, and any discord was resolved by discussion among the two reviewers (TY, NP) and the third reviewer (AC). Cohen’s kappa statistic was used to measure inter-rater reliability at each of the study selection steps. A secondary, separated search was conducted on 17 December 2019 to integrate most recent updates.

## Figures and Tables

**Table 1:** Findings from eligible studies assessing extracellular vesicles (EVs) in oral squamous cell carcinoma (OSCC) and oral potentially malignant disorders (OPMD) clinical cohort samples.

Study	Specimen Type	Markers	Assay	Findings
[13]	Salivary EVs	quantification morphology CD63 surface density	AFM, SMFS-CD63 mapping, Western blot analysis	- Increased vesicle size, irregular morphology, increased intervesicular aggregation observed in OSCC - Higher CD63 single vesicle density (by AFM) in OSCC
[14]	Salivary EVs	shotgun protein analysis	MS	- Proteome of OSCC EVs enriched in proteins involved in molecular transport and cellular growth and proliferation
[15]	Salivary exosomes	quantification CD81, CD9, CD63	NTA, AFM, TEM, ELISA, Western blotting	- Significantly higher concentration (*p* = 0.01) and size (*p* = 0.002) of nanoparticles in OSCC - lower expression of CD 81 (*p* = 0.032) in OSCC
[16]	Salivary EVs	microRNA	qPCR array; qPCR	- miR-302b-3p and miR-517b-3p expressed only OSCC-EVs vs. controls - miR-512-3p and miR-412-3p were up-regulated in OSCC-EVs vs. controls
[17]	Salivary exosomes	spectroscopy intensity ratios	Fourier-transform IR spectroscopy	- Increased (I1,404/I2,924) (*p* = 0.005), (I1,033/I1,072) (*p* = 0.024) and (I2,924/I2,854) (*p* = 0.026) in OSCC with sensitivity 100%, specificity 89%
[18]	Salivary exosomes	microRNA	microarray; qPCR	- 109 miRNA exhibited changes in their expression levels in OSCC EVs compared to normal controls - miR-24-3p was significantly higher in OSCC EVs in comparison to healthy controls (*p* < 0.05)
[19]	Salivary MVs and circulating MVs	Quantification; Annexin V	TEM; dynamic light scattering; CFSE labelling; flow cytometry	- Higher quantitative levels in OSCC (*p* < 0.05) vs. normal and benign ulceration - Annexin V+ decreased in high OSCC pathological grade (*p* < 0.01) and poorer survival (*p* < 0.05) - Higher quantitative levels of circulating MVs in OSCC (*p* < 0.001)
[20]	Plasma EVs	microRNA	microarray	- Exosomal fraction compared to free plasma shared all 9 upregulated and 6 of 7 downregulated microRNAs
[21]	Plasma EVs	Quantification; microRNA	NTA; qPCR	- Increased EV number (*p* < 0.001) and EV size (*p* < 0.05) in OSCC vs. controls - Increased miR-21, miR-27b and miR-27a increased in EV fraction vs. non-EV fraction in OSCC
[22]	Plasma EVs	CD63, Cav-1	immunocapture	- Non-significant decrease in CD63 post OSCC resection (*p* = 0.091) - non-significant increase in Cav-1 post OSCC resection (*p* = 0.237)
[23]	Serum exosomes	protein	LC-MS; MS; qPCR	- 37 differential proteins in OSCC with lymph node metastasis vs. healthy controls - 28 differentially expressed proteins in OSCC with lymph node metastasis vs. no lymph node metastasis esp. PF4V1, XCXL7, F13A1, ApoA1
**Oral Lichen Planus**				
	**Sample Type**	**Markers**	**Assay**	**Findings**
[24]	Salivary exosomes	microRNA	microarray; qPCR	- miR-4484 significantly upregulated in OLP
[25]	Plasma exosomes	microRNA	microarray	- Increased expression of miR-34a-5p, miR-130b-3p, and miR-29c-3p - Decreased miR-301b- 3p and miR-144-3p - miR-34a-5p correlated with increasing disease severity

Abbreviation List: AFM: Atomic force microscopy; CFSE: carboxyfluorescein succinimidyl ester; ELISA: enzyme linked immunosorbent assay; LC-MS: Liquid chromatography-mass spectrometry; MV: microvesicle; MS: mass spectrometry; NTA: nanoparticle tracking analysis; OSCC: oral squamous cell carcinoma; qPCR: quantitative polymerase chain reaction; TEM: transmission electron microscopy; SMFS: single molecular force spectroscopy.

**Table 2:** Findings from eligible studies assessing EVs in cell-based samples.

OSCC			
Study	Cell Type		Main Findings
	EVs Derived from	EVs Studied on	
	OSCC and Keratinocytes		
[26]	OSCC lines (OSC-3, OSC-4)	OSCC lines (OSC-3, OSC-4)	OSCC cell-derived exosomes promote source cell line proliferation, migration, and invasion in a dose dependent manner
[27]	OSCC lines (Cal-27, SCC9)	OSCC lines (Cal-27, SCC9)	OSCC—EVs produced under hypoxic conditions increased the migration and invasion of normoxic OSCC cells in a hypoxia-inducible factors—HIF1α and HIF-2α–dependent manner which was abrogated by miR-21 depletion
[28]	OSCC line (SQUU-B (metastatic))	OSCC line (SQUU-A (non-metastatic)	SQUU-B- exosomes s conferred metastatic ability to non-metastatic SQUU-A cells and reduced mRNA expression of cytokeratin 13
[29]	OSCC line (HOC313-LM (highly metastatic sub line))	OSCC line (HOC313-P (parent cell line))	HOC313-LM exosomes transferred oncogenic miR-343-3p and miR-1246 to HOC313-P cells and resulted in increase in cell motility and invasive ability
[30]	Cisplatin resistant OSCC cell lines (HSC3, SCC9)	Parental OSCC (HSC3 and SCC9)	EVs released from cisplatin-resistant OSCC cells transmit miR-21 to induce cisplatin resistance of OSCC cells
[31]	OSCC line (HSC3)	Oral keratinocytes (RT7)	OSCC derived EGFR-containing EVs were able to transform RT7 cells, effects of which were largely blocked by cetumixab
[32]	OSCC lines (Ca1 CALH2, SQCC/Y1) Premalignant buccal oral keratinocyte (SVpgC2a) Transformed malignant (SVFN9) normal oral keratinocyte lines (OH113, NK4, NOK368)	Primary normal oral keratinocytes	Exposure to OSCC-derived exosomes specifically modulated mRNA transcripts associated with matrix remodeling, cell cycle, differentiation, apoptosis, transcription and translation
	OSCC & Fibroblasts		
[33]	OSCC line (HSC3)	NOF	HSC3-exosomes lead to uptake by NOFs with resulting upregulation of expression of non-coding RNA Lnc-CAF
[23]	OSCC line (Cal-27)	HGFs (human gingival fibroblasts)	Cal-27 MVs were internalized increased levels of CAF markers (FAP and Tn-c) were isolated from HCFs, Cal-27 MV treated HGFs also showed increased glucose and lactate production with an increased expression of GLUT1, PDK1 and MCT4 but a decrease in CaV1
[34]	Primary OSCC CAFs	Primary OSCC keratinocytes	Exosome transfer from TGFβ signalling-competent fibroblasts increased transforming growth factor-beta receptor II (TBRII) levels and the TBRII signal transducing protein SMAD2 (but not SMAD3) phosphorylation in OSCC keratinocytes
[35]	Primary OSCC CAFs and adjacent tissue fibroblasts (AF)	OSCC lines (SCC25, SCC4)	CAF derived EVs increased C25(OTSCC) cell proliferation and migration compared with exosome depleted media or controls
[36]	Primary OSCC CAFs and matched NOFs	OSCC lines (Cal-27, SCC15)	CAF derived exosomes containing low or lentiviral plasmid restored expression of miR-34a-5p are transferred to OSCC cells
[37]	Primary CAFs and NOFs,	OSCC lines (HSC3, SAS, SCC15, SCC25)	CAF-EVS significantly increased the invasive, migration and apoptosis rate of HSC-3, SAS and SCC-15 but not SCC-25 with HSC-3 the most was most responsive to pooled CAF-EVs with deeper invasion, small tumour islands
[38]	Primary cancer associated fibroblasts; normal fibroblasts	OSCC line (Cal-27)	Significantly increased Cal-27 migration and invasion
[39]	Human dermal fibroblasts; normal keratinocytes	OSCC line (TR146)	Normal fibroblast and keratinocyte derived EVs suppressed OSCC proliferation but only at particular doses
	OSCC & Endothelial Cells		
[40]	OSCC cell lines (SQUU-A (non-metastatic), SQUU-B (metastatic))	endothelial cells (HUVECs, HDLECs)	Both OSCC-derived exosomes increased VEGFR2 expression in HUVECS; SQUU-B exosomes increased tube formation in HDLECs, both OSCC cell line derived exosomes stimulate expression of HDLEC mRNA expression of VEGFRs1-3 but only SQUU-B exosomes increased expression of VEFG-A,-C and -D
[41]	OSCC lines (Cal-27)	Endothelial cells (HUVECs)	Cal-27-MVs carrying Sonic hedgehog (Shh) protein significantly induce tube formation in HUVECS
[42]	OSCC lines (SCC15 AND HSC3)	Endothelial cells (HUVECs)	SSC15-EVs showed significant HUVEC tube formation, migration and increased apoptotic bodies vs. HSC3—EVs which significantly inhibited tube formation and proliferation; EVs derived from different OSCC cell lines are either pro-or anti angiogenic
[43]	OSCC lines (PCI-13, UMSCC47)	Endothelial cells (HUVECs)	PCI-13– exosomes caused significant increase in *VEGF* mRNA levels and *IGFBP-3* mRNA expression levels in the recipient cells; no significant changes after co-incubation of HUVECs with UMSCC47-derived exosomes
[44]	Metastatic OSCC subline (LN1-1) and parent line (OEC-M1)	Human dermal lymphatic endothelial cells (LECs)	LN1-1 derived EVs significantly increased migration and tube formation compared to incubation with parent cell
	OSCC & Immune Cells		
[12]	OSCC patient sera; T cells (Jurkat) and OSCC line (PCI-13)	T-blast cells, T cells (Jurkat)	OSCC serum MV fractions were FasL positive and induced DNA fragmentation, decreased the MMP potential or induced apoptosis of Jurkat cells, T blast cells or activated T lymphocytes
[21]	OSCC line (Cal-27) derived EVs	THP1 monocytes	Increase in miR-21-5p and activation of NF- κB suggesting pro-inflammatory, pro-tumorigenic shift
[45]	OSCC cell lines (SCC-25, Cal27)	NK cells	OSCC exosomes enhanced cytotoxicity of NK cells via the interferon regulatory factor 3 (IRF-3) pathway by delivery of that NF-κB-activating kinase-associated protein 1 (NAP1)
[46]	immortalized keratinocytes (HIOEC) leukoplakia cell line (Leuk1) OSCC cell lines (SCC25, Cal27)	Macrophages (THP-1 derived); healthy donor PBMCs	OSCC—exosomes but not HIOEC- or Leuk1- exosomes THP-1 and PBMCs derived macrophages into a M1 phenotype associated with tumor suppression
[47]	OSCC lines (Cal-27; SCC-29)	Primary γδ T cells	OSCC derived exosomes produced under normoxic conditions activated cytotoxicity of γδ T cells against these same oral cancer cell lines
[48]	OSCC line (SCC9, Cal-27), immortalized keratinocytes (HIOEC)	Macrophages (THP-1 derived), HBMCs	OSCC- exosome co-cultured macrophages showed higher expression levels of protein markers of M2 macrophage subtype: CD163, CD206, Arg-1, and IL-10; media of above cultured macrophages increased proliferation and invasive ability of OSCC cell lines with this effect abrogated by inhibition of miR-29a-3p
**OSCC and Mesenchymal Stem Cells**			
[49]	Primary mesenchymal stem cell (MSCs) from normal oral mucosa, dysplastic leukoplakia (LK) and OSCC	OSCC line (SCC-15); oral dysplasia line (DOK)	LK and OSCC mesenchymal stem cell derived exosomes both accelerated proliferation, invasion and migration of both SCC-15 and DOK cells
[50]	Primary human bone marrow mesenchymal stem cells	OSCC line (TCA 8113)	hBMSCs transfected with miR-101-3p-Cy3-derived exosomes donated miR-101-3p to OSCC cells repressing invasion and migration and reducing colony forming ability
**OPMD**			
**Study**	**Cell Type**		**Main Findings**
	**EVS Derived from**	**EVs Studied on**	
[51]	OLPPlasma-derived exosome from OLP patients	T lymphocytes (Jurkat)	T-cell proliferation and migration significantly increased with erosive LP-derived exosomes but not non-erosive LP exosomes

Abbreviation list: CAFs: cancer associated fibroblasts; HUVECs: human umbilical vein endothelial cells; HDLECs: human dermal lymphatic endothelial cells; NOFs: normal oral fibroblasts; OLP: oral lichen planus; OPMD: oral potentially malignant disorder; OSCC: oral squamous cell carcinoma; PBMC: peripheral blood mononuclear cells.

## Supplementary Materials

The following are available online at https://www.mdpi.com/1422-0067/21/4/1197/s1.
