# Supplementary material for: Extracellular Vesicles in Oral Squamous Cell Carcinoma and Oral Potentially Malignant Disorders: A Systematic Review
Source: Int J Mol Sci. 2020 Feb 11;21(4):1197. doi: 10.3390/ijms21041197 (PMC7072764; doi:10.3390/ijms21041197)
Supplement: Supplementary file 1 [file ijms-21-01197-s001.pdf]

Table S1. Search strategies applied to the four respective databases PubMed, Embase (Ovid), EBM Reviews (Ovid), and Web of Science (ISI).

Search #	Query
1	(extracellular vesicle* or exosom* or microvesicle* or ectosome* or shedding vesicle* or microparticle* or oncosome* or cell-derived microparticles or nanovesicle*).mp.
2	(oral or mouth or pharyn* or oropharyn* or throat* tongue or floor or palate or lingual or buccal or lip or labial or tonsil* or mucosa* or retromolar or cheek or gingiva or intra-oral or vermillion border).mp.
3	(pre-cancer* or cancer* or neoplasm* or malignan* or tumour or tumor or neoplasia or carcinoma or Leukoplakia or white patches or erythroplakia or red patches or erythroleukoplakia or precancer* or oral potentially malignant disorder or oral potentially malignant lesion or proliferative verrucous leukoplakia or dysplasia or premalignant or pre-malignant or lichen planus or oral submucous fibrosis or oral lupus erythematosus or actinic cheilitis or lichenoid or hyperplas* or ulcer or nodul* or metasta*).mp.
4	1 and 2 and 3
